# Serum VEGF-D level is correlated with renal dysfunction and proteinuria in patients with diabetic chronic kidney disease

**DOI:** 10.1097/MD.0000000000028804

**Published:** 2022-02-18

**Authors:** Thi Thuy Uyen Nguyen, Hyeongwan Kim, Yoon Jung Chae, Jong Hwan Jung, Won Kim

**Affiliations:** a Department of Histology, Embryology, Pathology and Forensic Medicine, Hue University of Medicine and Pharmacy, Hue University, Hue City, Viet Nam; b Department of Internal Medicine, Jeonbuk National University Medical School, Jeonju, Republic of Korea; c Department of Nursing, Kunjang University, Kunsan, Republic of Korea; d Division of Nephrology, Department of Internal Medicine, Wonkwang University School of Medicine and Hospital, Iksan, Republic of Korea; e Research Institute of Clinical Medicine of Jeonbuk National University-Biomedical Research Institute of Jeonbuk National University Hospital, Jeonju, Republic of Korea.

**Keywords:** albuminuria, chronic kidney disease, diabetic kidney disease, proteinuria, VEGF-D

## Abstract

Biomarkers associated with chronic kidney disease (CKD) may play a crucial role in the early diagnosis of diabetic kidney disease. However, there have been few reports published on serum vascular endothelial cell growth factor (VEGF)-D in patients with diabetic CKD. We divided patients with diabetic CKD into two groups: CKD 3–4 and CKD 5. In total, 42 patients with diabetic kidney disease and seven healthy controls without diabetes mellitus were enrolled in this study. An observational study was conducted to evaluate the serum VEGF-D levels and other clinical parameters in each group and to assess the relationship among these factors. The serum levels of VEGF-D were higher in the CKD 3–4 group and CKD 5 group than in the control group. However, there was no significant difference in serum levels of VEGF-D between CKD stage 3–4 group and CKD stage 5 group. Correlation analysis showed that serum VEGF-D was negatively correlated with estimated glomerular filtration rate but positively correlated with serum creatinine, urine albumin-to-creatinine ratio, and urine protein-to-creatinine ratio. Serum VEGF-D was a good biomarker in receiver operating characteristic analysis and independently associated with CKD stages in multiple linear regression analysis. Circulating VEGF-D was positively correlated with blood growth/differentiation factor-15, endostatin, and chemokine (C-X-C motif) ligand 16 levels. Serum VEGF-D levels were correlated with renal dysfunction, albuminuria, and proteinuria in patients with diabetic kidney disease. Elucidation of the role of VEGF-D as a biomarker requires further study.

## 1 Introduction

Diabetes mellitus (DM) is a well-known risk factor associated with cardiovascular diseases, including cerebral infarction, myocardial disease, and congestive heart disease.^[1]^ Chronic kidney disease (CKD) can also lead to cardiovascular complications. Moreover, cardiovascular disease is the main cause of morbidity and mortality in patients with CKD.^[^2^,^^3^^]^ Diabetic kidney disease is a leading cause of CKD and is strongly associated with the development of cardiovascular disease.^[^4^,^^5^^]^ The cardiovascular complications of diabetic kidney disease are primarily due to endothelial damage or dysfunction, including atherosclerotic changes and inflammation.^[6]^ Recently, progress has been made in identifying serum biomarkers associated with endothelial injury in patients with diabetic kidney disease.

Serum creatinine and albuminuria are very useful markers or indicators for identifying patients at risk of CKD development, including diabetic kidney disease, and they are clinical predictors of CKD progression.^[7]^ Moreover, serum creatinine and albuminuria in patients with diabetic kidney disease are widely used as indicators of the development of cardiovascular complications in patients with DM or diabetic kidney disease. However, new biomarkers for the progression of CKD, including diabetic kidney disease, are needed since serum creatinine and albumin levels can be influenced by various conditions, such as sex, age, inflammation, and nutrient status.^[8]^ According to previous literature, vascular endothelial dysfunction usually precedes a decrease in estimated glomerular filtration rate (eGFR) and development of albuminuria in patients with DM.^[^8^,^^9^^]^ Thus, serum biomarkers associated with endothelial injury may play a crucial role in the early detection of CKD progression in patients with diabetes.

Vascular endothelial growth factors (VEGFs) play important roles in the maintenance of vascular and lymphatic vascular endothelial cells under normal and pathological conditions. Vascular endothelial growth factor D (VEGF-D), a secreted glycoprotein belonging to the VEGF family, binds to vascular endothelial growth factor receptor (VEGFR)-2 and VEGFR-3. As VEGF-D binds both to VEGFR-2 and VEGFR-3, it increases angiogenesis and lymphangiogenesis through VEGFR-2 and VEGFR-3, respectively.^[^10^,^^11^^]^ It has been demonstrated that high serum levels of VEGF-D are associated with atrial fibrillation, ischemic stroke and heart failure.^[^12^,^^13^^]^ Recently, increased VEGF-D values have been linked to all-cause mortality in patients with coronary artery disease.^[14]^ It has been shown that high serum levels of VEGF-C, another member of the VEGF family, are associated with a decrease in renal function in CKD patients.^[15]^ However, serum levels of VEGF-D in diabetic patients with CKD have not been reported previously.

To identify the relationship between serum VEGF-D levels and renal function, we measured the levels of serum VEGF-D in patients with diabetic CKD. We also evaluated the relationships between serum VEGF-D levels and clinical parameters including albuminuria, proteinuria, and other biomarkers related to kidney function in patients with diabetic kidney disease.

## 2 Materials and methods

### 2.1 Study patients and criteria

This was a single-center, observational study carried out at the Jeonbuk National University Hospital for patients with diabetic CKD. The study was conducted in accordance with the guidelines of the Declaration of Helsinki and approved by the Ethical Committee of Jeonbuk National University Hospital (protocol number: 2018-12-029-01; date of approval: December 31, 2019). Informed consent was obtained from all the subjects involved in the study.

We defined type 2 diabetes as fulfilling at least one of the following criteria that was used in a previous study:

1. self-reported type 2 DM;
2. physician-diagnosed type 2 DM according to laboratory data;
3. fasting blood glucose ≥126 mg/dL (two tests); or
4. HbA1c >6.5%.^[16]^

To analyze the serum levels of VEGF-D in healthy people and patients with diabetic CKD stage 3 or 4 and CKD stage 5, we divided the CKD patients into two groups: CKD 3–4 stage (CKD 3-4) and CKD stage 5 (CKD 5). We enrolled a total of 49 patients, including seven controls, 28 CKD 3–4 patients, and 14 CKD 5 patients. All enrolled patients were above 20 years of age and the 7 controls had DM. Twenty-eight patients with diabetic CKD stage 3 or 4 were defined by the Chronic Kidney Disease Epidemiology Collaboration (CKD-EPI) based on an eGFR of 30 to 59 mL/min/1.73 m^2^ and 15 to 29 mL/min/1.73 m^2^, respectively, and 14 patients with diabetic CKD stage 5 were defined as CKD-EPI eGFR < 15 mL/min/1.73 m^2^. In addition, diabetic kidney disease in this study was defined as a condition when patients with DM type 2 simultaneously had the following: CKD-EPI eGFR < 60 mL/min/1.73 m^2^ and urine albumin-to-creatinine ratio (UACR) > 30 mg/g or urine protein-to-creatinine ratio (UPCR) > 150 mg/g.

In this study, we excluded patients with CKD undergoing hemodialysis or peritoneal dialysis, patients with previous histories of dialysis and transplantation, diabetic CKD patients with eGFR < 60 mL/min/1.73 m^2^ resulting from acute renal dysfunction due to various clinical conditions, and DM patients with eGFR > 60 mL/min/1.73 m^2^, showing results of UACR or UPCR, which fulfilled the diagnostic criteria for diabetic kidney disease. All enrolled patients with diabetic kidney disease were predialytic CKD patients.

### 2.2 Measurement of serum VEGF-D, growth/differentiation factor-15, endostatin and chemokine (C-X-C motif) ligand 16

Blood samples (∼7 mL) were drawn from patients in the supine position to investigate serum levels of VEGF-D in patients with diabetic CKD and healthy patients. Immediately following collection and centrifugation, serum and urine samples were transported to the Biobank of Jeonbuk National University Hospital and stored at −80°C until analysis. The serum for this study was provided by the Biobank of Jeonbuk National University Hospital. The serum levels of VEGF-D, growth/differentiation factor-15 (GDF15), endostatin, and chemokine (C-X-C motif) ligand 16 (CXCL16) were measured using the Luminex 200 System (Luminex, TX) and the Human Magnetic Luminex Assay Kit (R&D Systems, MN).

### 2.3 Measurement of serum and urine creatinine and calculation of eGFR

Serum and urine creatinine levels were measured using the kinetic rate Jaffe method. We used the CKD-EPI creatinine equation to calculate the eGFR, considering race as “non-black.”^[17]^

### 2.4 Statistical analyses

R language version 3.3.3 (R Foundation for Statistical Computing, Vienna, Austria) and T&F program ver. 1.7 (YooJin BioSoft, Korea) was used for all statistical analyses. Data are expressed as mean ± standard deviation (SD) or median (Q1–Q3 quartile) for continuous variables. When variables were normally distributed, the mean difference between two sample groups was determined using Student's *t* test or Welch's *t* test. One-way ANOVA was performed for comparison of more than two groups. For non-normally distributed variables, the Mann–Whitney *U* test or Kruskal–Wallis *H* test was used accordingly. Post hoc analysis was performed using Bonferroni's algorithm. For categorical variables, data were expressed as sample number and percentage, N (%), and the chi-square test or Fisher's exact test was used for comparison of sample proportions as appropriate. Pearson correlation and Spearman correlation coefficients were computed to analyze the correlation between continuous variables. Predictors and outcome variables were identified and analyzed using multiple linear regression. Non-normally distributed variables were logarithmically transformed. Receiver operating characteristic (ROC) curve analysis was performed to estimate the prediction accuracy of the continuous values. The cutoff value was selected, where the summation of sensitivity and specificity was maximized. A *P-*value < .05 was considered as significant.

## 3 Results

### 3.1 Baseline characteristics

The enrolled patients were divided into three groups: 28 patients with diabetic kidney disease in the CKD 3-4 group, 14 patients with diabetic kidney disease in the CKD 5 group, and 7 controls. All patients with diabetic kidney disease were predialyzed. The baseline characteristics of the 49 participants are presented in Table 1. The mean age of patients with diabetic kidney disease in the CKD 3–4 group and CKD 5 group was 63.8 ± 2.2 and 62.7 ± 3.7 years, respectively. There were no significant differences in age or sex distribution between the groups. Although there was a tendency for increased blood pressure in patients in the CKD 5 group compared with those in the CKD 3–4 group or the control group, there was no statistically significant difference in mean values of systolic blood pressure and diastolic blood pressure between the groups (Table 1).

**Table 1:** Baseline characteristics of enrolled patients.

Variables	Control group	CKD 3–4 group	CKD 5 group	*P*
Number	7 (14.3)	28 (57.1)	14 (28.6)	
Age (year)	51.6 ± 2.9	63.8 ± 2.2	62.7 ± 3.7	.06
Male, n (%)	3 (42.9)	14 (50.0)	10 (71.4)	.401
Serum creatinine (mg/dL)	0.6 (0.5–0.8)	2.2 (1.6–2.9)	5.3 (4.3–6.1)	< .001
eGFR (mL/min/1.7m^2^)	109.6 (98.5–119.0)	29.2 (15.3–37.9)	9.8 (7.6–13.7)	< .001
BMI (kg/m^2^)	24.9 (23.1–26.6)	25 (21.2–27.3)	23.4 (20.2–26.9)	.705
UPCR (mg/g)	50.0 (30.0–80.0)	821.3 (280.4–4628.0)	4212.9 (2626.0–5343.0)	< .001
UACR (mg/g)	12.4 (6.7–14.4)	627.2 (85.4–3506.0)	2252.4 (1096.5–5235.0)	< .001
SBP (mm Hg)	120 (115–130)	127 (120–141)	133 (114–146)	.342
DBP (mm Hg)	70 (65–75)	68 (63–76)	71 (64–81)	.490
Total cholesterol (mg/dL)	174.0 (163.0–207.0)	149.0 (112.7–186.3)	176.5 (126.5–228.3)	.137
Triglycerides (mg/dL)	265 (135.5–580.5)	118.0 (86.3–144.0)	232 (141.7–286.7)	.251
LDL cholesterol (mg/dL)	36 (32–51)	39 (32–49)	33 (27–44)	.499

Data are mean ± standard deviation (SD) or median (inter-quartile range). BMI = body mass index, DBP = diastolic blood pressure, eGFR = estimated glomerular filtration rate, LDL = low density lipoprotein, SBP = systolic blood pressure, UACR = urine albumin-to-creatinine ratio, UPCR = urine protein-to-creatinine ratio.

### 3.2 Blood level of VEGF-D increased in CKD patients

Serum VEGF-D levels ranged from 3 to 150 pg/mL among the subjects in this study. VEGF-D concentrations in the CKD 3–4 group or CKD 5 groups were significantly increased compared to those in the control group (Table 2). The CKD stage 5 patients did not show a significant difference in VEGF-D blood levels compared to CKD 3–4 patients. VEGF-D was not detected in urine with the method used in this study.

**Table 2:** Comparison of serum levels of VEGF-D according to CKD stage.

	Control group (N = 7)	CKD 3-4 group (N = 28)	CKD 5 group (N = 14)	*P* ^1^	*P* ^2^	*P* ^3^
Serum VEGF-D (pg/mL)	30 (16–39)	55 (39–84)	61 (42–91)	< .01	< .01	.669
Urine VEGF-D (pg/mg)	Undetectable	Undetectable	Undetectable			

Data are median (inter-quartile range). CKD = chronic kidney disease, VEGF-D = vascular endothelial growth factor-D, *P* ^1^value = comparison between control group and CKD 3–4 group, *P* ^2^ value = comparison between control group and CKD 5 group, *P* ^3^ value = comparison between CKD 3–4 group and CKD 5 group.

### 3.3 Negative correlation of VEGF-D with eGFR

To determine whether increases in serum VEGF-D levels were associated with serum creatinine and eGFR, we performed the correlation analysis. As shown in A and B, serum VEGF-D levels were positively associated with serum creatinine levels and negatively associated with eGFR. These findings suggest that elevated VEGF-D concentrations are closely associated with changes in renal function.

**Figure 1:**
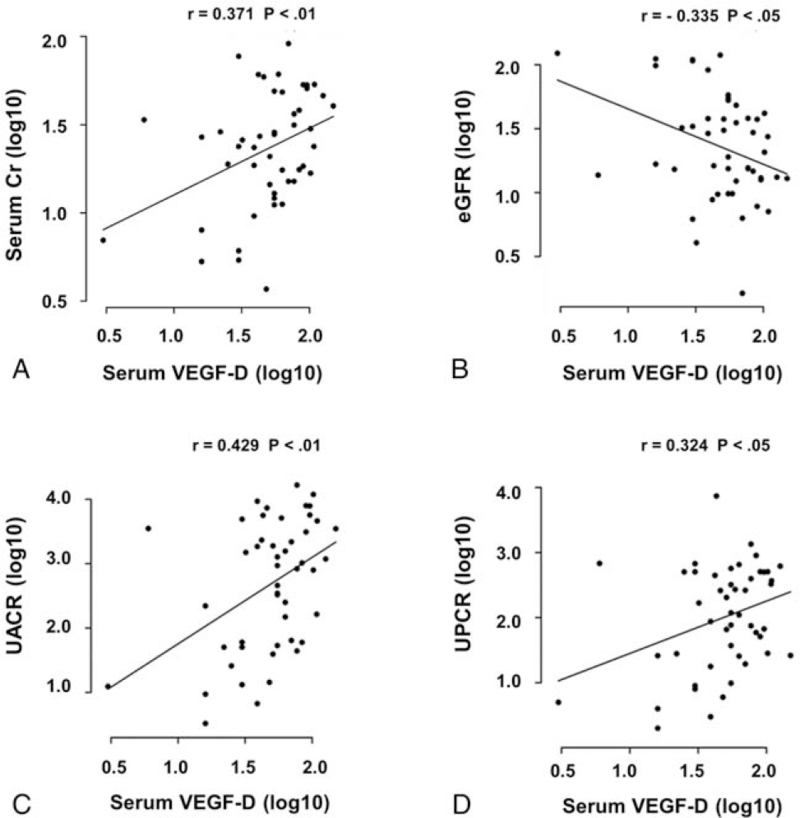
Correlation of serum VEGF-D level with serum creatinine (A), estimated glomerular filtration rate (eGFR) (B), UACR (C), and UPCR (D). Serum VEGF-D level of healthy volunteer and CKD patient is plotted against serum creatinine, eGFR, UACR, and UPCR. Correlation analysis was evaluated using Pearson correlation method, *r*-value and the *P*-value are given.

### 3.4 Associations of serum VEGF-D with albuminuria and proteinuria

We assessed the correlation between serum VEGF-D levels with UPCR and UACR. Correlation analysis showed that there were statistical associations between serum VEGF-D levels and UPCR and UACR (C and D).

### 3.5 ROC analysis

**Figure 2:**
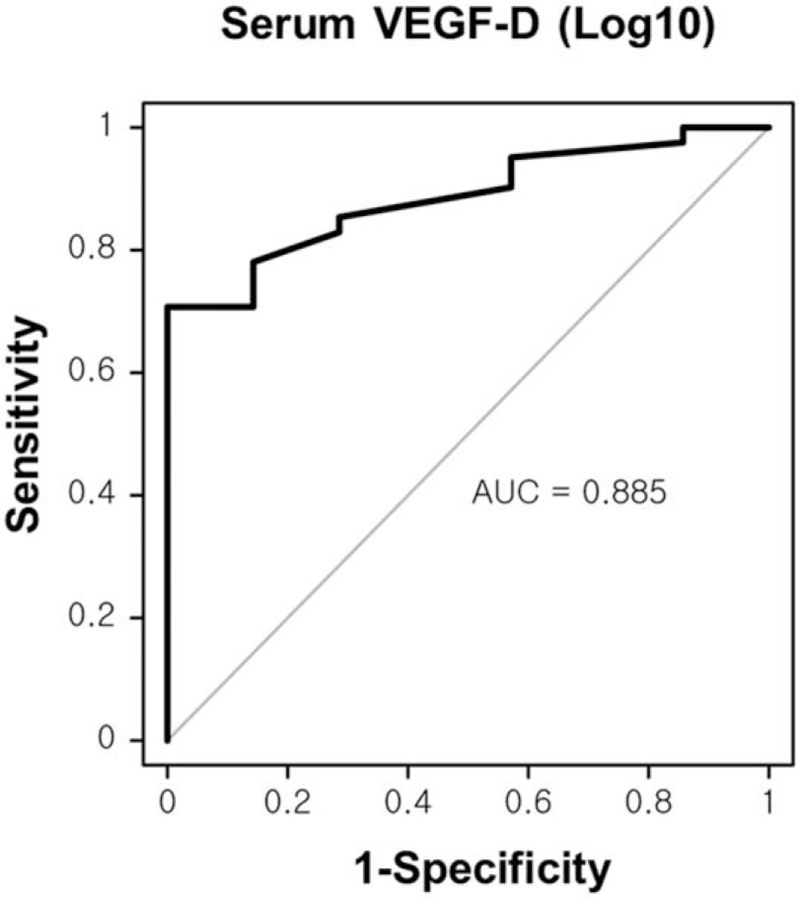
Receiver operating characteristic (ROC) curve of serum VEGF-D for CKD (AUC = 0.885, 95% confidence interval: 0.783–0.987, *P* = .001).

The results of the ROC analyses are presented in Figure 2 (AUC = 0.885, 95% confidence interval: 0.783–0.987, *P* = .001). This curve shows that serum VEGF-D is an excellent biomarker for discriminating CKD from healthy subjects.

### 3.6 Stages 3 to 4 and stage 5 were independently associated with serum VEGF-D level

We performed multivariate linear regression to assess the relationship between a model of independent variables (predictors) and the dependent variable. The variables including stages 3 to 4 group, stage 5, age, sex, BMI, total cholesterol, cerebral infarction, and myocardial infarction were selected as independent variables of serum VEGF-D. The analysis revealed that the stages 3 to 4 group, stage 5 groups were independently associated with serum VEGF-D levels (Table 3).

**Table 3:** Multiple linear regression analysis of predictors for serum vascular endothelial growth factor (VEGF)-D.

Independent variable	Standardized coefficients (β)	*P*
Stages 3–4 group vs control group	0.34	.02
Stage 5 group vs control group	0.366	.002
Age	0.005	.232
Sex	−0.046	.624
BMI	0.001	.946
Total cholesterol	0.00	.946
Cerebral infarction	−0.205	.069
Myocardial infarction	0.034	.743

The analysis used stages 3–4 group vs control group, stage 5 group vs control group, age, sex, BMI, total cholesterol, cerebral infarction and myocardial infarction as independent variables of logarithmically transformed VEGF-D. Adjusted *R* square = 0.237. BMI = body mass index.

### 3.7 Circulating VEGF-D level was positively correlated with blood GDF15, endostatin, and CXCL16

**Figure 3:**
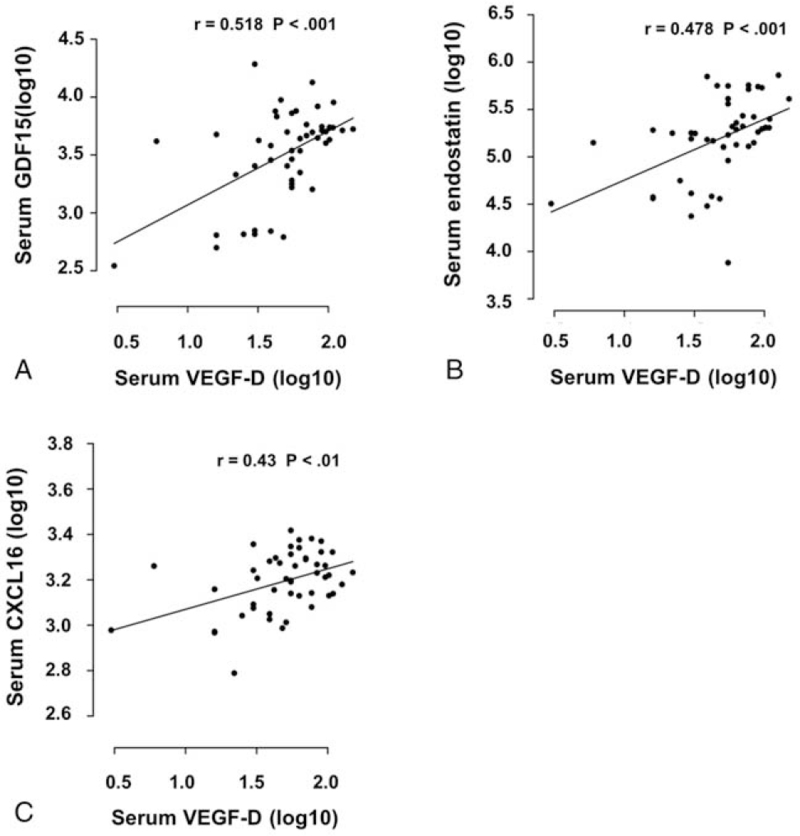
Correlation of serum VEGF-D level with serum GDF15 (A), endostatin (B), and CXCL16 (C). Circulating VEGF-D level of healthy volunteer and CKD patient is plotted against serum GDF15, endostatin and CXCL16. Correlation analysis was evaluated using Pearson correlation method; *r*-value and *P*-value are provided.

Recently, several biomarkers, including serum GDF15, endostatin, and CXCL16, were increased in CKD patients and they were associated with renal function.^[^18^–^20^]^ Therefore, we measured these biomarkers and evaluated the relationship between circulating VEGF-D and blood GDF15, endostatin, and CXCL16. Our study showed that serum GDF15, endostatin, and CXCL16 levels were significantly increased in patients with diabetic CKD (Table 4). We also found positive correlations between circulating VEGF-D and GDF15, endostatin, and CXCL16 (Fig. 3).

**Table 4:** Comparison of serum GDF15, endostatin and CXCL16 levels according to CKD stage.

	Control group (N = 7)	CKD 3-4 group (N = 28)	CKD 5 group (N = 14)	*P* ^1^	*P* ^2^	*P* ^3^
Serum GDF15 (pg/mL)	642 (500–695)	3906 (2309–5106)	6168 (4886–7941)	<.001	<.001	<.01
Serum endostatin (pg/mL)	36,020 (30,196–37,639)	185,215 (135,793–255,247)	229,824.5 (153,032–543,264)	<.001	<.001	.210
Serum CXCL16 (pg/mL)	971 (938–1189)	1635 (1357–1898)	1927 (1492–2353)	<.001	<.001	.055

Data are expressed as median (inter-quartile range). GDF15 = growth/differentiation factor-15, CXCL16 = Chemokine (C-X-C motif) ligand 16, CKD = chronic kidney disease, *P* ^1^ value = comparison between control group and CKD 3–4 group, *P* ^2^ value = comparison between control group and CKD 5 group, *P* ^3^ value = comparison between CKD 3–4 group and CKD 5 group.

## 4 Discussion

VEGF-C and VEGF-D are known growth factors involved in lymphangiogenesis.^[21]^ These two growth factors play important roles in angiogenesis and lymphangiogenesis. Recently, Sahutoglu et al^[15]^ reported an increase in blood VEGF-C levels in patients with chronic kidney disease. Therefore, changes in the concentration of VEGF-C in CKD should be further investigated. Due to the close relationship between VEGF-D and VEGF-C, we suggest that there is a significant association between VEGF-D and CKD. Recently, it has been reported that blood VEGF-D levels are increased in patients with lymphangioleiomyomatosis.^[22]^ However, changes in serum VEGF-D levels are not well known in CKD patients.

Up-to-date, there is no definite report on the impact of serum VEGF-D on the progression of CKD in patients with diabetic kidney disease, and this is the first time that circulating VEGF-D levels have been evaluated in patients with diabetic CKD. In this study, the serum level of VEGF-D increased in patients with CKD and was also correlated with a decline in eGFR. Our data suggest that serum VEGF-D levels are a potential biomarker for diabetic CKD patients. We also tried to measure the urine levels of VEGF-D biomarkers in diabetic CKD patients. However, the urine concentration of VEGF-D was very low and it could not be measured because it was outside the range of measurement with our method.

We noted both similarities and differences between our findings and those of previous studies on serum VEGF-D concentrations.^[^23^,^^24^^]^ Similar to Young et al, we found that the baseline VEGF-D concentration in the control group was very low (<39 pg/mL). However, in contrast with our findings, Young et al showed that serum VEGF-D levels were significantly higher in patients with lymphangioleiomyomatosis (>600 pg/mL) than in those with CKD (39–90 pg/mL).^[24]^ Differences between our results and those reported in previous studies might be a result of the usage of different measurement methods and patient groups.

It has been shown that GDF15 is related to cardiovascular disorders such as endothelial dysfunction, heart failure, and atherosclerosis in DM.^[25]^ High GDF15 levels are related to renal function decline in patients with type 1 diabetes.^[26]^ Circulating levels of endostatin are significantly increased and strongly correlated with the severity of CKD.^[^27^,^^28^^]^ In patients with type 2 diabetes, serum endostatin levels can be a predictive marker of kidney disease progression and mortality.^[29]^ Additionally, a previous study indicated a significant association between blood endostatin levels and cardiovascular events in CKD.^[30]^ Serum levels of CXCL16 have been reported to be significantly associated with kidney function and cardiovascular disease.^[18]^ In this study, we evaluated the levels of GDF15, endostatin, and CXCL16 in the serum of patients with diabetic CKD and found that serum levels of these biomarkers were significantly increased in CKD patients. This finding is consistent with the results of previous studies. In addition, our results demonstrated a positive relationship between serum VEGF-D levels and these biomarkers. Since GDF15, endostatin, and CXCL16 are suggested to be biomarkers of CKD, our data suggest that VEGF-D may have a role as a biomarker in diabetic CKD.

This study did not show the exact mechanism related to increased serum levels of VEGF-D in diabetic patients with decreased renal function. This study had several limitations. The total number of enrolled patients was small. However, because there were increases in serum VEGF-D levels, we could demonstrate the use of serum VEGF-D as a predictor of CKD progression in patients with diabetic kidney disease through this clinical study. A more dedicated study with a larger number of patients could confirm serum VEGF-D as a stronger biomarker for identifying prognosis, early diagnosis, and disease progression in diabetic CKD. Our major limitation is that we did not determine whether VEGF-D is a biomarker of kidney function or diabetes. Thus, further studies to compare the level of VEGF-D in healthy controls, patients with diabetes and CKD, patients with diabetes without CKD, and CKD patients without diabetes are needed. This study was not a prospective observational study that identified changes in VEGF-D according to the decline of renal function in patients with diabetic kidney disease. Therefore, prospective observational studies are needed in the future.

In this study, serum VEGF-D levels in the CKD 5 group were significantly higher than those in the control group. However, serum levels were not significantly higher than those in the CKD 3-4 group (Table 2). Our data suggest that serum VEGF-D is correlated with renal dysfunction, albuminuria, and proteinuria in patients with diabetic kidney disease. In addition, a study with a larger number of patients and a well-structured design could validate the use of serum VEGF-D levels as statistically significant predictors or indicators of eGFR reduction in patients with diabetic kidney disease.

In conclusion, our data suggest that serum VEGF-D is closely related to a decline in renal function, albuminuria, and proteinuria in patients with diabetic chronic kidney disease.

## Acknowledgments

The biospecimens and data used in this study were provided by the Biobank of Jeonbuk National University Hospital, a member of the Korea Biobank Network, which is supported by the Ministry of Health, Welfare, and Family Affairs. All samples derived from the Korea Biobank Network were obtained with informed consent under institutional review board-approved protocols. We would like to thank Editage (www.editage.co.kr) for English language editing.

## Author contributions

This paper is a result of the cooperation and contributions of all authors. Conceptualization, W.K. and J.H.J.; Methodology, W.K. and H.K.; Software, T.T.U.N. and W.K.; Validation, W.K. and H.K.; Formal Analysis, Y.J.C., T.T.U.N, H.K., and W.K.; Investigation, H.K., Y.J.C., J.H.J., and W.K.; Writing – Original Draft Preparation, Y.J.C., T.T.U.N. and H.K.; Writing – Review & Editing, T.T.U.N, H.K., and W.K.; Supervision, W.K. and J.H.J.; Project Administration, W.K.; Funding Acquisition, W.K.. All authors read and approved the final version of the manuscript.

**Conceptualization:** Jong Hwan Jung, Won Kim.

**Formal analysis:** Thi Thuy Uyen Nguyen, Hyeongwan Kim, Yoon Jung Chae, Won Kim.

**Funding acquisition:** Won Kim, Jong Hwan Jung.

**Investigation:** Yoon Jung Chae, Jong Hwan Jung, Won Kim.

**Methodology:** Hyeongwan Kim, Won Kim.

**Project administration:** Won Kim.

**Software:** Thi Thuy Uyen Nguyen, Won Kim.

**Supervision:** Jong Hwan Jung, Won Kim.

**Validation:** Hyeongwan Kim.

**Writing – original draft:** Thi Thuy Uyen Nguyen, Hyeongwan Kim, Yoon Jung Chae, Won Kim.

**Writing – review & editing:** Thi Thuy Uyen Nguyen, Hyeongwan Kim, Won Kim.
